# Patterns of instrumental activities of daily living and association with predictors among community-dwelling older women: A latent class analysis

**DOI:** 10.1186/s12877-017-0557-6

**Published:** 2017-07-21

**Authors:** Jeongok Park, Young Joo Lee

**Affiliations:** 1 Mo-Im Kim Nursing Research Institute, College of Nursing, Yonsei University, 613 College of Nursing, Yonsei-ro 50, Seodaemun-gu, Seoul 03722 South Korea; 2 College of Nursing, Daegu Catholic University, 33, Duryugongwon-ro 17-gul, Nam-gu, Daegu, 42472 South Korea

**Keywords:** Aged, Instrumental activities of daily living, Latent class analysis, Women

## Abstract

**Background:**

The purpose of this study was to classify patterns of instrumental activities of daily living (IADL) among community-dwelling older women, to examine difference in characteristics among the classes, and to explore predictors of class membership.

**Methods:**

This study was a secondary analysis of nationwide data from the 2014 Actual Living Condition of the Elderly and Welfare Need Survey. A total of 10,451 individuals aged 65 years or older were interviewed for the 2014 dataset, but we only selected the female participants (*n* = 6095) for this study. For statistical analyses, latent class analysis was applied to identify different latent classes of IADL and then the effects of predictors on IADL patterns were analyzed by using multinomial logistic regression.

**Results:**

The 5-class model was the best fit for the data. The size of class 1was the biggest (*n* = 5093, 83.6%), followed by class 5 (*n* = 401, 6.6%), class 3 (*n* = 308, 5.1%), class 2 (*n* = 181, 3.0%), and class 4 (*n* = 113, 1.8%). The largest class had total independency on all items of IADL. In the multinomial regression, members in the classes 2, 3, 4 and 5 were significantly more likely to have older age and decreased cognitive status compared with the class of total independency on all items of IADL (class1).

**Conclusions:**

The predictors of the classes identified in this study can be used for tailored and targeted interventions to increased old adults’ independency on IADL.

## Background

As people live longer, not only individuals but also health care providers are interested in how to be old “successfully.” One of the major constituents of successful aging, as defined by psychosocial or biomedical experts as well as older adults, is high levels of independent physical and cognitive functioning [1]. Dependence is the main impact factor on health and quality of life, not only for elderly people but also for the caregiver and relatives. An instrumental activities of daily living (IADL) scale, which is based on tasks that allow independent living [2], has been used to evaluate older people’s functional capabilities. The IADL scores relate to tasks that require sufficient capacity to make decisions as well as greater interaction with the environment [3].

Previous studies have found that total IADL scores are significantly associated with the female gender [4], increased age [4, 5], cognitive impairment [6], education [4, 7], depression [5], comorbidities [5, 8], and residential area [7]. To evaluate older adults’ functional status, the majority of studies have used IADL as a measure of functional capacity. That is, they have simply added up the items of limited IADL, which has implied that they are equivalent in their importance. However, in previous studies, a multidimensional structure of IADL items has been found through exploratory factor analysis [9]. In addition, some studies have found an association of some of the IADL items with risk factors [10]. Although the multidimensionality of IADL has been suggested, there has been little investigation of the characteristics of elderly people who have limited IADL, specifically older women who has higher prevalence rates of IADL compared to men [11]. Therefore, classifying older women’s IADL capability and identifying risk factors by the class could help in the comprehensive understanding of their health problems and for developing targeted interventions for the classes who have different limitations of IADL.

The aims of this study were to classify patterns of individuals within a sample of community-dwelling older women, to examine differences in characteristics among the IADL classes, and to explore predictors of class membership.

## Methods

This study was a secondary analysis of nationwide data from the 2014 Actual Living Condition of the Elderly and Welfare Need Survey [12]. A total of 10,451 individuals aged 65 years or older (male 4356; female 6095) were interviewed for the 2014 dataset, but we selected only the female subjects for this study. The Institutional Review Board of Y University approved our protocol for this study (IRB no. 2015–0045).

The primary variable for this study was IADL, which was measured using the Korean IADL scale [13]. The question, “Did you need any help to perform this activity over the last week?” was used to collect the data. A total of 10 items (grooming, housekeeping, food preparation, laundry, responsibility for own medications, handling money, going out, shopping, phone use, and mode of transportation) was evaluated to estimate the IADL score. Subjects were asked to rate their condition on the first seven items as “total independence,” “partial dependence,” or “total dependence.” The last three items were rated as “total independence,” “partial dependence,” “total dependence,” or “complete inability.” The total IADL score ranges from 10 to 33, and a lower score indicates a higher level of independence. When we classified the class, the responses of “total dependence” and “complete inability” were considered as “total dependence” for the last three items.

The demographic data analyzed in this study were age, marriage, educational level, place of residence, body mass index, and cognitive status. The Korean version of the Mini-Mental State Examination-Dementia Screening (MMSE-DS) was used to evaluate cognitive status. The total possible score ranges from 0 to 30, and a lower score indicates a lower level of cognitive function. Subjects’ score of MMSE-DS was categorized into normal or decreased by their age and educational level [12]. Self-reported comorbidities of hypertension, stroke, diabetes mellitus (DM), arthritis, urinary incontinence (UI), depression, and experience of falls were also included in our analyses.

Data analyses were conducted using SPSS version 23 (IBM, Armonk, NY) and poLCA package of R program within SPSS Statistics for latent class analysis (LCA). LCA was applied to identify different latent classes of IADL. This is a person-centered method that can be used to categorize those who perform similarly in indicator patterns within the same subgroup [14]. First, it was to fit a LCA model with the categorical indicators only. The indicator in this study was IADL score, which was considered as ordinal variable. The optimal model was selected according to the Bayesian information criterion (BIC) value; a smaller BIC value represents a better model fit. Second, the class memberships were assigned to the participants based on maximum posterior probability method derived from the LCA model. After the class was assigned, the average IADL scores were calculated for each assigned class. Finally, the effects of predictor on class variable were analyzed using multinomial logistic regression. Except for IADL, other variables such as age, BMI, education, residence, comorbidities, experience of falls, and cognitive status were used as auxiliary variables.

## Results

The subjects’ characteristics (*n* = 6095) are described in Table 1. Their mean age was 74.6 years (*SD* = 6.88). Sixty-three percent of subjects had normal BMI and approximately 32% were overweight or obese. About 44% of subjects had a spouse and 42% were illiterate. Approximately three-quarters of subjects lived in a metropolitan city. Regarding the subjects’ comorbidities, hypertension (60%) was the most prevalent health problem and arthritis was the second most prevalent (49%). About 31% of subjects had experience of falls. Regarding cognitive status, 29% of subjects showed decreased cognitive function. The mean IADL score was 11.1 (*SD* = 3.21). About 18.5% (*n* = 1126) of subjects reported receiving some help to perform IADL from someone. Among them 90% of respondents were receiving help from their family and only 17% were receiving in-home services provided by long-term care insurance, such as home-visit care, day and night care, or short-term respite care.

**Table 1:** Characteristics of the subjects. (*n* = 6095)

Variables	*N* (%)	Mean ± *SD*	Range
Age (years)		74.6 ± 6.88	65–105
65–79	4604 (75.5)	71.4 ± 4.42	
≥ 80	1491 (24.5)	84.3 ± 3.67	
BMI^a^		23.7 ± 3.40	12.8–49.1
Underweight (<18.5)	315 (5.2)	17.3 ± 1.01	
Normal (18.5–24.9)	3839 (63.0)	22.2 ± 1.71	
Overweight/obese (≥25.0)	1940 (31.8)	27.5 ± 2.26	
Marriage			
Having a spouse	2655 (43.6)		
No spouse^b^	3440 (56.4)		
Education			
Illiterate	2578 (42.3)		
Elementary school	2097 (34.4)		
≥ Middle school	1421 (23.3)		
Residence			
Metropolitan	4636 (76.0)		
Nonmetropolitan	1460 (24.0)		
Comorbidities			
Hypertension	3671 (60.2)		
Stroke	384 (6.3)		
DM	1400 (23.0)		
Arthritis	2992 (49.1)		
UI	586 (9.6)		
Depression	271 (4.4)		
Experience of falls			
Yes	1861 (30.5)		
No	4234 (69.5)		
MMSE^a^ (sum of scores)		22.6 ± 5.00	0–30
Normal	4203 (69.0)	24.7 ± 3.39	
Decreased	1763 (28.9)	17.6 ± 4.56	
IADL^a^ (sum of scores)		11.1 ± 3.21	10–33

*SD* standard deviation, *BMI* body mass index, *DM* diabetes mellitus, *UI* urinary incontinence, *MMSE* Mini-Mental State Examination, *IADL* instrumental activities of daily living

^a^Excluding missing data, ^b^no spouse: single, divorced, or widowed

**Fig. 1:**
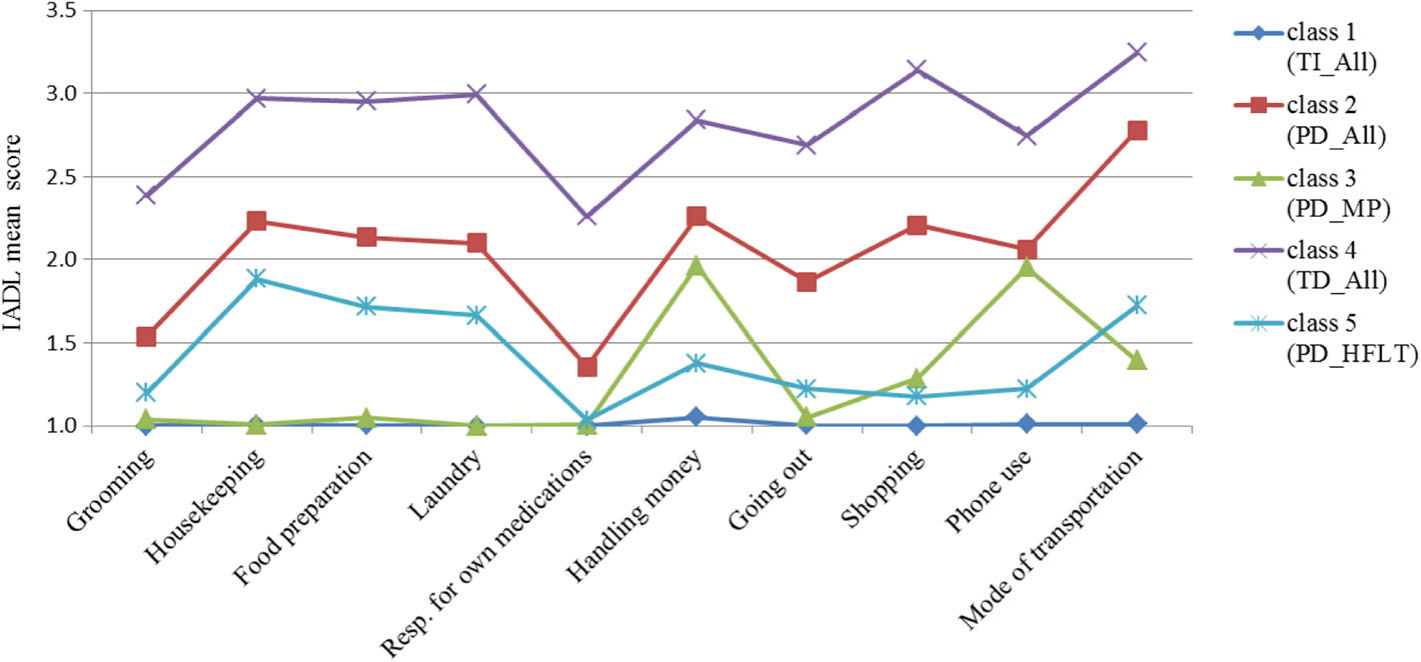
Distribution of the mean instrumental activities of daily living scores for each of the items by class. IADL: instrumental activities of daily living, resp. for own medications: responsibility for own medications, TI-All: total independence for all instrumental activities of daily living items, PD-All: partial dependence for all instrumental activities of daily living items, PD-MP: partial dependence for the instrumental activities of daily living items of handling money and phone use, TD-All: total dependency for all instrumental activities of daily living items, PD-HFLT: partial dependence for the instrumental activities of daily living items of housekeeping, food preparation, laundry, and mode of transportation

Based on the minimal BIC value, a five-class model was the best fit for the data. Class 1 was the largest (*n* = 5093, 83.6%), followed by Class 5 (*n* = 401, 6.6%), Class 3 (*n* = 308, 5.1%), Class 2 (*n* = 181, 3.0%), and Class 4 (*n* = 113, 1.8%). Figure 1 shows the IADL profiles for each class, with mean scores plotted for each item by class. Class 1 had total independence for all IADL items (TI-All). Class 2 had partial dependence for all IADL items (PD-All) and Class 3 had partial dependence for the IADL items of handling money and phone use (PD-MP). Class 4 had total dependence for all IADL items (TD-All). Class 5 had partial dependence for most basic self-care IADL items, such as housekeeping, food preparation, laundry, and mode of transportation (PD-HFLT).

The frequencies of the subjects’ characteristics are presented in Table 2 for each class. Regarding age, 82% of Class 1 was from 65 to 79 years old while, in contrast, only 32% of Class 2 was in that range. Having a spouse (47%) was highest in Class 1. Class 2 had the highest proportion of being underweight (13%), and the most prevalent DM (34%) and depression (9%). The rate of illiteracy (*n* = 263, 85%) was highest in Class 3. In Class 4, having a stroke (25%) and UI (27%) were most prevalent and the rate of decreased cognitive status was the highest. Arthritis (57%) and experience of falls (50%) were the most prevalent in Class 5.

**Table 2:** Relationships between class membership and the subjects’ characteristics. Unit: *n* (%)

Variables	Class 1	Class 2	Class 3	Class 4	Class 5	χ^2^	*p*
	5093 (83.6)	181 (3.0)	308 (5.1)	113 (1.8)	401 (6.6)		
Age (years)							
65–79	4164 (81.8)	58 (32.2)	141 (45.6)	37 (33.0)	204 (51.0)	679.1	< .001
≥ 80	929 (18.2)	122 (67.8)	168 (54.4)	75 (67.0)	196 (49.0)		
BMI							
Underweight	219 (4.3)	24 (13.3)	19 (6.2)	13 (11.5)	40 (10.0)	72.1	< .001
Normal	3194 (62.7)	112 (61.9)	207 (67.2)	75 (66.4)	251 (62.6)		
Overweight/obese	1679 (33.0)	45 (24.9)	82 (26.6)	25 (22.1)	110 (27.4)		
Marriage							
Having a spouse	2380 (46.7)	57 (31.7)	77 (25.0)	27 (23.9)	113 (28.2)	130.3	< .001
No spouse^a^	2712 (53.3)	123 (68.3)	231 (75.0)	86 (76.1)	287 (71.8)		
Education							
Illiterate	1934 (38.0)	119 (66.1)	263 (85.1)	63 (56.3)	199 (19.6)	352.2	< .001
Elementary	1842 (36.2)	33 (18.3)	44 (14.2)	32 (28.6)	146 (36.4)		
≥ Middle school	1317 (25.9)	28 (15.6)	2 (0.6)	17 (15.2)	56 (14.0)		
Residence							
Metropolitan	3929 (77.2)	140 (77.3)	165 (53.4)	94 (83.2)	308 (76.8)	93.9	< .001
Nonmetropolitan	1163 (22.8)	41 (22.7)	144 (46.6)	19 (16.8)	93 (23.2)		
Comorbidities							
Hypertension	3016 (59.2)	123 (68.0)	191 (61.8)	72 (63.7)	270 (67.3)	16.0	.003
Stroke	262 (5.1)	27 (14.9)	21 (6.8)	28 (25.0)	46 (11.5)	118.9	< .001
DM	1102 (21.6)	61 (33.7)	71 (23.0)	32 (28.6)	135 (33.7)	44.8	< .001
Arthritis	2463 (48.4)	90 (49.7)	163 (52.9)	47 (41.6)	228 (56.9)	15.1	.004
UI	419 (8.2)	35 (19.3)	35 (11.4)	30 (26.8)	67 (16.7)	93.2	< .001
Depression	225 (4.4)	16 (8.9)	6 (1.9)	5 (4.4)	19 (4.7)	13.0	.011
Experience of falls							
Yes	1409 (27.7)	87 (48.3)	114 (37.0)	49 (43.4)	202 (50.4)	135.9	< .001
No	3684 (72.3)	93 (51.7)	194 (63.0)	64 (56.6)	199 (49.6)		
Cognitive status^b^							
Normal	3771 (74.2)	51 (36.4)	155 (52.4)	10 (17.2)	216 (55.7)	277.5	< .001
Decreased	1314 (25.8)	89 (63.6)	141 (47.6)	48 (82.8)	172 (44.3)		

*BMI* body mass index, *DM* diabetes mellitus, *UI* urinary incontinence

^a^No spouse: single, divorced, or widowed, ^b^measured using the Mini-Mental State Examination

In the multinomial regression (Table 3), compared with the TI-All class, members of all other classes were significantly more likely to be older and to have decreased cognitive status. Members of the PD-All class had approximately twice the odds of underweight BMI (odds ratio [OR] = 2.21, 95% confidence interval [CI] 1.24–3.96), DM (OR = 1.83, 95% CI 1.23–2.72), UI (OR = 1.89, 95% CI 1.14–3.13), depression (OR = 2.28, 95% CI 1.17–4.43), and experience of falls (OR = 1.98, 95% CI 1.39–2.83) compared with the TI-All class. Furthermore, the odds of illiteracy (OR = 3.06, 95% CI 1.70–5.52) and stroke (OR = 3.12, 95% CI 1.83–5.31) were three times greater compared with the TI-All class. Members of the PD-MP class had 45 times greater odds of being illiterate (OR = 44.75, 95% CI 12.08–165.81) compared with the TI-All class. In addition, they were significantly less likely to live in a metropolitan city (OR = 0.51, 95% CI 0.40–0.66) and more likely to have experience of falls (OR = 1.36, 95% CI 1.05–1.76) compared with the TI-All class. Members of the TD-All class were more likely to live in a metropolitan city (OR = 2.18, 95% CI 1.05–4.55) compared with the TI-All class. They had about seven times greater odds of having a stroke (OR = 7.23, 95% CI 3.76–13.87) and were twice as likely to have DM (OR = 2.11, 95% CI 1.18–3.77) compared with the TI-All class. Members of the PD-HFLT class had about twice the odds of being underweight (OR = 1.86, 95% CI 1.25–2.75), having a stroke (OR = 2.34, 95% CI 1.64–3.32), DM (OR = 1.93, 95% CI 1.52–2.45), UI (OR = 1.79, 95% CI 1.32–2.44), and experiencing a fall (OR = 2.39, 95% CI 1.92–2.97) than the TI-All class.

**Table 3:** Multinomial logistic regression analyses of instrumental activities of daily living classes^a^. Unit: Odds ratio (95% CI)

Variables	Class 2 (PD-All)	Class 3 (PD-MP)	Class 4 (TD-All)	Class 5 (PD-HFLT)
Age (≥80 years)	7.53 (5.00, 11.34)^***^	3.08 (2.35, 4.02)^***^	5.30 (2.88, 9.76)^***^	3.47 (2.73, 4.41)^***^
BMI (underweight)	2.21 (1.24, 3.96)^**^	0.88 (0.51, 1.50)	1.87 (0.71, 4.94)	1.86 (1.25, 2.75)^**^
(overweight/obese)	1.06 (0.71, 1.60)	0.89 (0.67, 1.19)	1.26 (0.70, 2.29)	0.89 (0.69, 1.13)
Education (illiterate)	3.06 (1.70, 5.52)^***^	44.75 (12.08,165.81)^***^	1.64 (0.77, 3.52)	1.47 (1.05, 2.07)^*^
(elementary)	1.15 (0.60, 2.20)	10.58 (2.79, 40.06)^**^	0.74 (0.32, 1.73)	1.42 (1.01, 1.98)^*^
Residence (metropolitan)	1.39 (0.91, 2.12)	0.51 (0.40, 0.66)^***^	2.18 (1.05, 4.55)^*^	1.13 (0.87, 1.47)
Comorbidities				
Hypertension (yes)	1.18 (0.80, 1.74)	1.01 (0.77, 1.31)	0.97 (0.54, 1.74)	1.14 (0.90, 1.44)
Stroke (yes)	3.12 (1.83, 5.31)^***^	1.40 (0.86, 2.27)	7.23 (3.76, 13.87)^***^	2.34 (1.64, 3.32)^***^
DM (yes)	1.83 (1.23, 2.72)^**^	1.26 (0.93, 1.69)	2.11 (1.18, 3.77)^*^	1.93 (1.52, 2.45)^***^
Arthritis (yes)	0.70 (0.48, 1.01)	0.91 (0.70, 1.17)	0.93 (0.53, 1.63)	1.18 (0.95, 1.48)
UI (yes)	1.89 (1.14, 3.13)^*^	1.41 (0.95, 2.09)	2.06 (0.98, 4.30)	1.79 (1.32, 2.44)^***^
Depression (yes)	2.28 (1.17, 4.43)^*^	0.53 (0.23, 1.21)	0.10 (0.00, 5.10)	1.07 (0.65, 1.77)
Experience of falls (yes)	1.98 (1.39, 2.83)^***^	1.36 (1.05, 1.76)^*^	1.24 (0.71, 2.19)	2.39 (1.92, 2.97)^***^
Cognitive status (decreased)	4.49 (3.12, 6.45)^***^	2.30 (1.79, 2.96)^***^	13.21 (6.53, 26.73)^***^	2.11 (1.69, 2.63)^***^

*PD-All* partial dependency for all instrumental activities of daily living items, *PD-MP* partial dependence for the handling money and phone use items of instrumental activities of daily living, *TD-All* total dependence for all instrumental activities of daily living items, *PD-HFLT* partial dependence for the housekeeping, food preparation, laundry, and mode of transportation items of instrumental activities of daily living, *BMI* body mass index, *DM* diabetes mellitus, *UI* urinary incontinence

^a^Reference group: Class 1, ^*^ *p*-value < .05, ^**^ *p*-value < .01, ^***^ *p*-value <.001

## Discussion

In the current study, the average IADL score of the older women was 11.1 and the percentage of TI-All was 83.6%. The rate of total independence for the IADL in Korean older women is higher compared with a study of older women in Spain [3] in which the rate was 56%. Regarding the rate of total dependence for the IADL, the present study and Millan-Calenti et al. (2010) found 1.8% and 2.9%, respectively. This finding indicates that the majority of the Korean older women living in communities are still able to perform IADL independently.

It was clear that subjects in the PD-All, PD-MP, TD-All, and PD-HFLT classes were more likely to be older in age and to have cognitive impairment. Previous studies have also found that there is a significant association between the deterioration of IADL and advanced age [3, 15]. Similarly, other authors [6, 16] found that cognitive impairment was an independent predictor of functional loss measured by IADL.

The current study found that both the PD-All and PD-HFLT classes shared many predictors such as stroke, DM, UI, experience of falls, and underweight status. The subjects in these two classes have physical disability in common. The association between IADL decline and UI is well documented in the literature [17, 18]. Specifically, women with physical disability, such as mobility or functional limitations, are reported to have UI significantly more than women without disability [17], which was also found in the present study. There is evidence that training in mobility and toileting skills, and prompted voiding for older adults with UI were effective treatment [19, 20]. Therefore, physical training to improve toileting and education regarding prompted voiding for older adults and their caregivers is needed for the PD-All and PD-HFLT classes. Regarding the prevention of falls, it is known that multifactorial approaches that combine multiple interventions, such as home modifications, education about health and safety, medication management, vision management, gait and balance training, and exercise, are effective for elderly people having difficulties with IADL [21]. Thus, some of these interventions could be offered to the subjects and caregivers in these classes.

According to the literature, inadequate nutrient intake and BMI are predictors of the severity of ADL disability [22, 23]. The present study demonstrated a finding consistent with this; being underweight was a significant predictor in both the PD-All and PD-HFLT classes. Because the subjects in these classes were not independent for the IADL item of food preparation, customized interventions such as a home-delivered meal service [24] and/or comprehensive nutritional counseling involving active participation [25] could be applied to the subjects and their caregivers.

Depression was an independent predictor in the PD-All class. It is known that the need for assistance with IADL is associated with depression in community-dwelling older adults [10, 26]. In detail, Chiu et al. found that, among the IADL items, being unable to go shopping increased the risk of depression in older adults [10]. Similarly, the subjects in the PD-All class that were partially dependent for shopping had significantly higher odds of depression compared to the subjects in the TI-ALL class in the current study.

Illiteracy and living in nonmetropolitan areas were independent predictors in the PD-MP class. It is well established in the literature that educational level is associated with the item of managing money in elderly women [3]. Regarding the IADL item of phone use, the question, “Can you find phone numbers, and make phone calls and answer the phone without any help?” was used to collect the data. The subjects who were illiterate could not perform the activity of finding phone numbers in a telephone book. Thus, little wonder illiteracy was a significant predictor of the PD-MP class. Living in nonmetropolitan areas was also a significant predictor of this class. Thus, when performing health education or interventions for older adults living in nonmetropolitan areas, health care providers need to consider their health literacy. In addition, educational courses could be offered in elementary school or in the Korean language for elderly people living in rural areas.

The TD-All class was the smallest class in the current study. A possible explanation for this is that older adults who need significant assistance with all of the IADL items, who may be bedbound or wheelchair-bound, are less likely to live in their homes. Stroke was the strongest predictor in this class. It is known that stroke limits independent IADL [27]. Furthermore, the subjects of this class were more likely to live in a metropolitan city compared to those in the TI-All class. A similar result was found in a previous study in which 72% of stroke patients lived in urban areas [28]. The reason for this result could be due to differences in family support, living arrangements, and social support between metropolitan and nonmetropolitan areas [10]. To reduce the dependency for IADL of the subjects in this class, interventions such as task-oriented training for stroke patients [27] could be offered. Additionally, strategies to prevent or reduce caregiver burden and emotional problems in partners of stroke patients [29] need to be developed and offered to the caregivers.

This study has several limitations. First, because of the secondary data analysis, the selection of variables to examine the predictors of IADL was limited. Although, in the literature, diseases, such as dementia [30, 31] and Parkinson’s disease [32, 33], household income [7], social participation [34], and hospital admission [3, 6] have been found to be significant factors associated with older adults’ functional status, this information was not available for the analysis. Therefore, future research is needed to determine the association of these factors with older adults’ IADL. Second, because this study had a cross-sectional design, the causal relationship between the factors and IADL cannot be determined. Intervention or longitudinal studies are needed to verify the effects of the predictors on IADL for each class. Lastly, because the maximum posterior probability method without classification uncertainty adjustment was used for identifying underlying latent categorical variable in the current study, the latent class variable could lose its meaning as the latent variable measured by the indicator variables [35].

Despite these limitations, our results have important implications. The current findings highlight that the majority of community-dwelling older women is independent for the IADL. We found that older women who are partially dependent for the physical items of IADL were significantly more likely to have had a stroke, DM, UI, depression, falls, and being underweight. Thus, health care providers should pay attention to older women who are dependent for the physically related items of IADL with co-existing health problems. Such an approach could help to prevent these health problems or to detect them early and thus maintain or improve their optimal health status. We also found that the majority of caregivers of older women who need assistance to perform the IADL were family members. This finding implies that community-based homemaker services, and providing or renting assistive devices that enhance older women’s independence to perform IADL, could be appropriate interventions [26]. In addition, allied health workers, including community health providers, are often in close contact with older adults and they could play an important role in identifying the need for assistance with IADL because they provide home-visit services for them [26].

## Conclusions

In conclusion, five distinct classes of IADL among community-dwelling older women were identified. The largest class was totally independent for all items of IADL. The other four classes were associated with older age and cognitive impairment compared with the class with total independence for IADL. The predictors of the classes identified in this study can be used for tailored and targeted interventions to increase older adults’ independence for IADL.

## Abbreviations

BMI: Body mass index
DM: Diabetes mellitus
IADL: Instrumental activities of daily living
MMSE: Mini-Mental State Examination
PD-All: Partial dependency for all instrumental activities of daily living items
PD-HFLT: Partial dependence for the housekeeping, food preparation, laundry, and mode of transportation items of instrumental activities of daily living
PD-MP: Partial dependence for the handling money and phone use items of instrumental activities of daily living
TD-All: Total dependence for all instrumental activities of daily living items
UI: Urinary incontinence
